# Human *Taenia martis* Neurocysticercosis, Switzerland

**DOI:** 10.3201/eid2912.230697

**Published:** 2023-12

**Authors:** Valentin K. Steinsiepe, Marie-Therese Ruf, Marco Rossi, Claudia Fricker-Feer, Danijela Kolenc, Brigitte Suter Buser, Maura Concu, Andreas Neumayr, Ulf C. Schneider

**Affiliations:** Cantonal Hospital of Lucerne, Lucerne, Switzerland (V.K. Steinsiepe, M. Rossi, C. Fricker-Feer, D. Kolenc, U.C. Schneider);; Swiss Tropical and Public Health Institute, Allschwil, Switzerland (M.-T. Ruf, M. Concu, A. Neumayr);; University of Basel, Basel, Switzerland (M.-T. Ruf, M. Concu, A. Neumayr)

**Keywords:** taenia, neurocysticercosis, parasites, tapeworms, parasitic diseases, central nervous system, zoonoses, Switzerland

## Abstract

Neurocysticercosis is almost exclusively caused by *Taenia solium* tapeworms. We describe a case of neurocysticercosis in Switzerland caused by infection with *Taenia martis*, the marten tapeworm, and review all 5 published cases of human infection with the marten tapeworm. In epidemiologically nonplausible cases of neurocysticercosis, zoonotic spillover infections should be suspected.

Neurocysticercosis is a zoonotic, parasitic, central nervous system infection almost exclusively caused by the larvae of *Taenia solium*, the pork tapeworm (*1*). In a few exceptional cases, neurocysticercosis in humans is not caused by *T. solium* but by other zoonotic *Taenia* species, representing rare spillover infections from distant ecologic niches (Appendix Table). In this article, we describe a rare case of *T. martis* neurocysticercosis in a woman in Switzerland.

A woman 55 years of age sought care at the emergency department of the Cantonal Hospital of Lucerne (Lucerne, Switzerland) because of a 3-week history of progressive transient numbness and convulsions of her left hand. Her medical history was unremarkable. Clinical examination revealed disorientation to time, a pronator drift of the left arm, hypoesthesia of the left extremities, and a tactile neglect toward the left side. A comprehensive metabolic panel and a complete blood count showed no major abnormalities. Computed tomography of the brain revealed a 12 × 14 mm mass in the right postcentral gyrus with strong ring-enhancement and perifocal edema (Appendix Figure 1). We started the patient on levetiracetam and admitted her for further investigation.

Results of computed tomography of the thorax and abdomen were unremarkable. Magnetic resonance imaging (MRI) (Appendix Figure 2) showed no restriction on diffusion-weighted imaging. After starting the patient on dexamethasone, we removed the lesion through a right parietal craniotomy. Postoperative MRI confirmed complete resection. We gradually discontinued dexamethasone and discharged the patient 6 days after the operation.

The specimen consisted of a cyst with a thick wall and with no macroscopically discernible content. Histologic analysis revealed a singular membrane, compatible with a helminthic parasite, and an abscessing inflammation at the border (Figure). Results of immunohistochemical tests, eubacterial 16S PCR, and whole-genome sequencing were negative, as was serologic screening for *Echinococcus* spp. However, Western blot for cysticercosis, which uses *T. solium* IgG (LDBIO Diagnostics, https://www.ldbiodiagnostics.com), revealed a weak band pattern suggestive of an infection with *T. solium*. We contacted the Swiss Tropical and Public Health Institute, which raised the issue of missing epidemiologic plausibility, given that the patient had never traveled to a *T. solium*–endemic area. The possibility of zoonotic spillover infection was considered and MRI of the whole spine, ocular ultrasound, and stool investigations recommended to exclude additional lesions and taeniasis. We analyzed the resected neurocysticercus by using a pan-helminthic PCR and sequencing of the cestode- and nematode-specific cytochrome c oxidase subunit 1 (*cox1*) gene (*2*), which revealed 100% sequence identity with *Taenia martis*, the marten tapeworm (GeneBank accession no. OQ536306).

**Figure:**
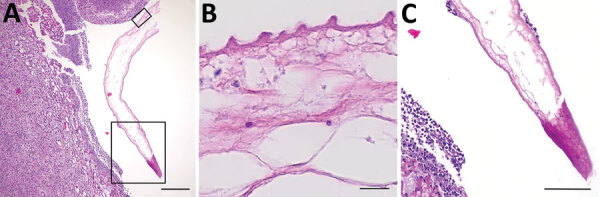
Histologic sections of resected *Taenia martis* metacestode from a patient in Switzerland. A) Cross-section through the excised tissue revealed strong infiltration surrounding a structure suggestive of parasitic origin. Boxed areas are shown in a higher magnification in panels B and C. B, C) The metacestode’s cyst wall, showing the warty appearance (B) characteristic of tapeworm metacestodes’ tegument (*3*,*4*). Hematoxylin and eosin stain. Scale bars indicate 200 μM (A), 10 μM (B), and 100 μM (C).

Because of the unclear date of infection, the unknown proliferation rate of the parasite in humans, and the possibility of undetectable lesions, we treated the patient with albendazole and praziquantel, analogous to treatment for *T. solium* neurocysticercosis, assuming similar susceptibility of the parasite (*3*). Three months after the operation, the patient showed no neurologic deficits and no new focal seizures.

The definitive hosts of *T. martis* tapeworms are martens and other mustelids. *T. martis* tapeworms have been found in the intestines of 36% of stone martens (*Martes foina*) in southwest Germany but also parasitize other mustelids (*4*,*5*). Stone martens occur throughout Europe and Central Asia. The natural intermediate hosts of *T. martis* tapeworms are small rodents, which develop cysticerci in the pleural and peritoneal cavities. The infection in this patient suggests an accidental fecal–oral transmission, although it remains unclear when and how she came into contact with marten droppings.

Diagnosed human infections with *T. martis* tapeworms are limited to 5 cases reported from Germany and France (Table; Appendix Figure 3) (*6*–*10*). Those cases were 2 peritoneal infections, 2 eye infections, and 1 central nervous system infection, all with single lesions occurring in immunocompetent women. Of note, an increased susceptibility to various *Taenia* spp. tapeworms dependent on sex and hormone status has been described in animals (*5*). All 6 patients, including ours, lived in rural villages, 5 (including ours) grew their own vegetables, and at least 3 (including ours) had frequent marten sightings around their homes. Similar to what other authors reported, we did find cross-reactivity with a *T. solium*–specific assay.

**Table:** Overview of published cases of human *Taenia martis* infections

Authors (year of publication)	Country	Patient age, y/sex	Chief complaint	Location of lesion	False-positive serologic assays	Treatment
Eberwein et al. (2013) (*6*)	Germany	43/F	Flashing lights and paracentral scotoma	Left eye	No	Albendazole 400 mg 2×/d, dexamethasone 20 mg/d for 8 d, surgical excision
Brunet et al. (2015) (*7*)	France	44/F	Right hemiparesis and aphasia	Left temporal lobe	*T. solium*	Surgical excision, praziquantel 50 mg/kg for 15 d, albendazole 15 mg/kg for 1 mo, corticosteroids 1 mg/kg) (sic)
Koch et al. (2016) (*8*)	Germany	70/F	Drop in visual acuity	Left eye	No	Surgical excision, albendazole 400 mg 2×/d for 7 d
Rudelius et al. (2017) (*9*)	Germany	36/F	Ascites	Pouch of Douglas	*Echinococcus granulosus*	Surgical excision, albendazole 400 mg 2×/d for 4 weeks
Mueller et al. (2020) (*10*)	Germany	24/F	Lower abdominal pain and dysmenorrhea	Pouch of Douglas	*E. multilocularis, T. solium, Dirofilaria immitis.*	Surgical excision; antiparasitic treatment refused by patient
Steinsiepe et al. (2023) (this report)	Switzerland	55/F	Focal epileptic seizures	Right parietal lobe	*T. solium*	Surgical excision, praziquantel 50 mg/kg/d for 14 d, albendazole 15 mg/kg/d for 28 d, corticosteroids

All but 1 of the patients described in the published cases received antiparasitic treatment; the only untreated case was in a patient who refused medical treatment (*10*). None of the published cases reported recurrence or emergence of additional lesions.

Neurocysticercosis may be caused by *Taenia* spp. other than *T. solium* tapeworms. Cases in which an infection with *T. solium* is epidemiologically not plausible should be investigated for zoonotic spillover infections (in Central Europe, specifically infection with *T. martis* should be considered). Such cases are probably underrepresented in the literature, given the high prevalence of stone martens, their high infection rate with *T. martis* tapeworms, and the possibility of false positives of available, cross-reactive *T. solium*– and *Echinococcus* spp.–specific serologic assays. If adequate material is available, a panhelminthic PCR with *cox1* sequencing is highly recommended.

Appendix Additional information about human *Taenia martis* neurocysticercosis, Switzerland.
