# Supplementary material for: Human Taenia martis Neurocysticercosis, Switzerland
Source: Emerg Infect Dis. 2023 Dec;29(12):2569–72. doi: 10.3201/eid2912.230697 (PMC10683805; doi:10.3201/eid2912.230697)
Supplement: Appendix — Additional information about human Taenia martis neurocysticercosis, Switzerland. [file 23-0697-Techapp-s1.pdf]

Article DOI: https://doi.org/10.3201/eid2912.230697

EID cannot ensure accessibility for supplementary materials supplied by authors. Readers who have difficulty accessing supplementary content should contact the authors for assistance.

Human *Taenia martis* Neurocysticercosis, Switzerland

Appendix

Appendix Table. Zoonotic *Taenia* spp. to be considered as rare causes of human neurocysticercosis (*1*,*2*)

Taenia species	Natural definite host	Natural intermediate host	Known geographic range of the parasite
T. multiceps	Dog and other canids	Sheep	Worldwide
T. serialis	Dog and other canids	Rabbit, hare	North America, Europe, Africa
T. brauni	Dog and other canids	Rabbit, hare	Africa
T. glomerata	Dog and other canids	Rabbit, hare	Africa
T. crassiceps	Dog, fox, cat	Rodents	North America, Europe, Russia
T. martis	Marten and other mustelids (also rarely canids, wild cat)	Rodents	North America, Europe, Russia

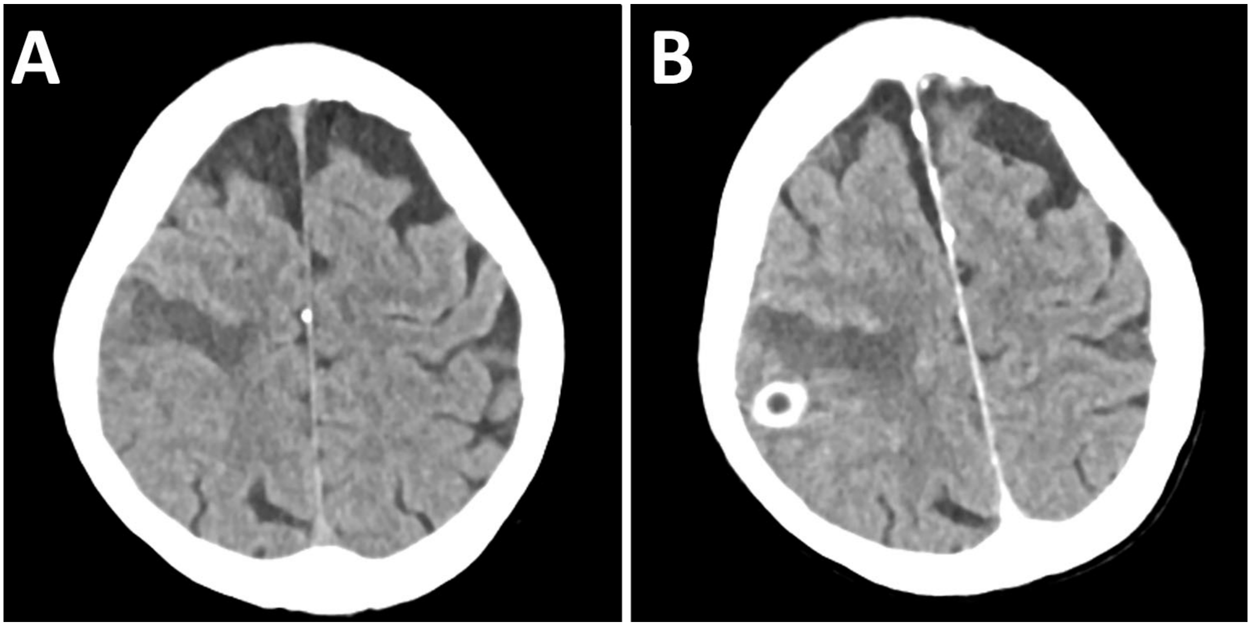

Appendix Figure 1. Axial CT of the head showing a ring-enhancing lesion in the right postcentral gyrus. A) Noncontrast. B) Contrast enhanced.

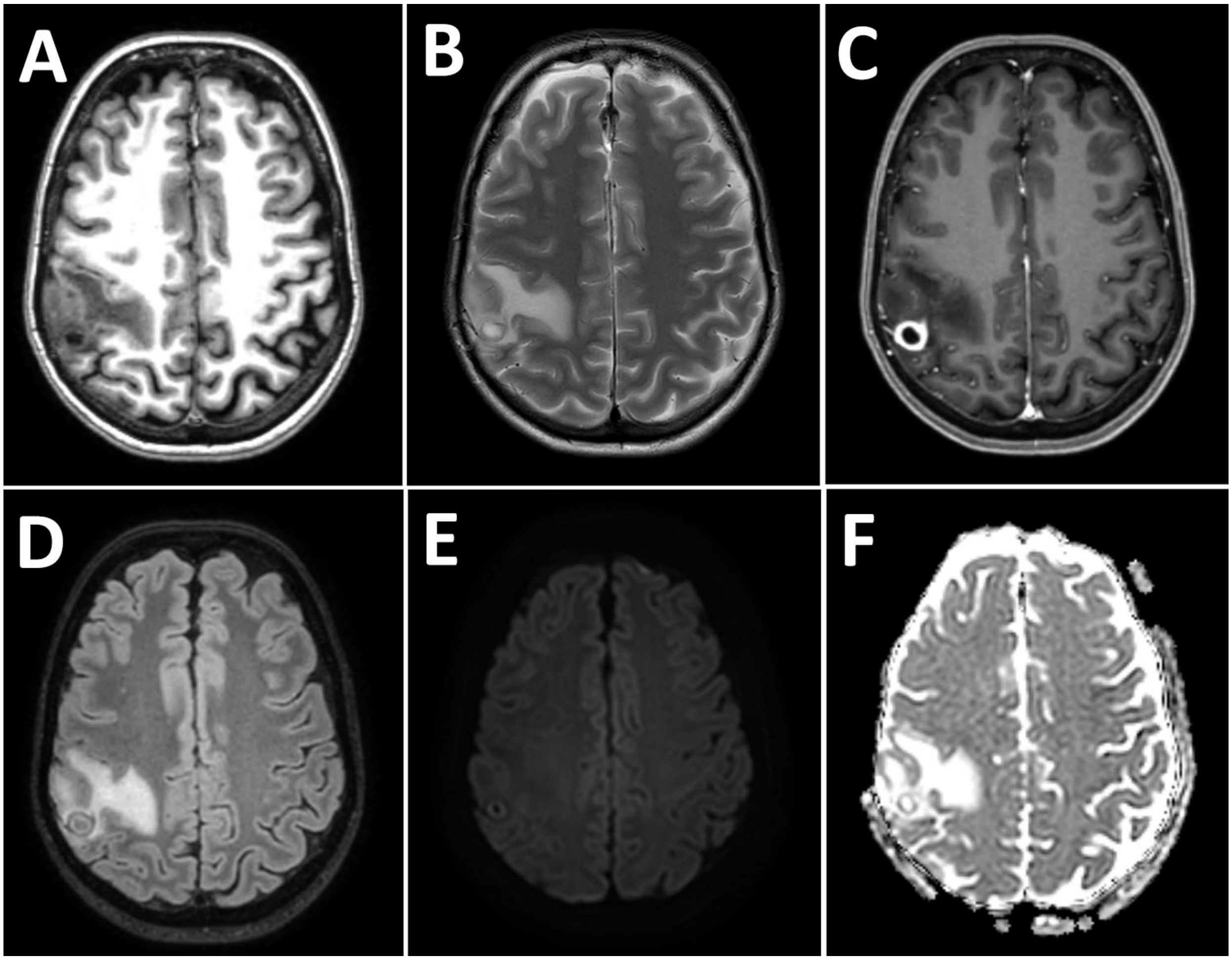

Appendix Figure 2. Axial magnetic resonance imaging of the larval cyst in the right postcentral gyrus. A) T1. B) T2. C) T1 enhanced. D) Fluid attenuated inversion recovery (FLAIR). E) Diffusion-weighted imaging (DWI B1000) (B1,000). F) Apparent diffusion coefficient (ADC).

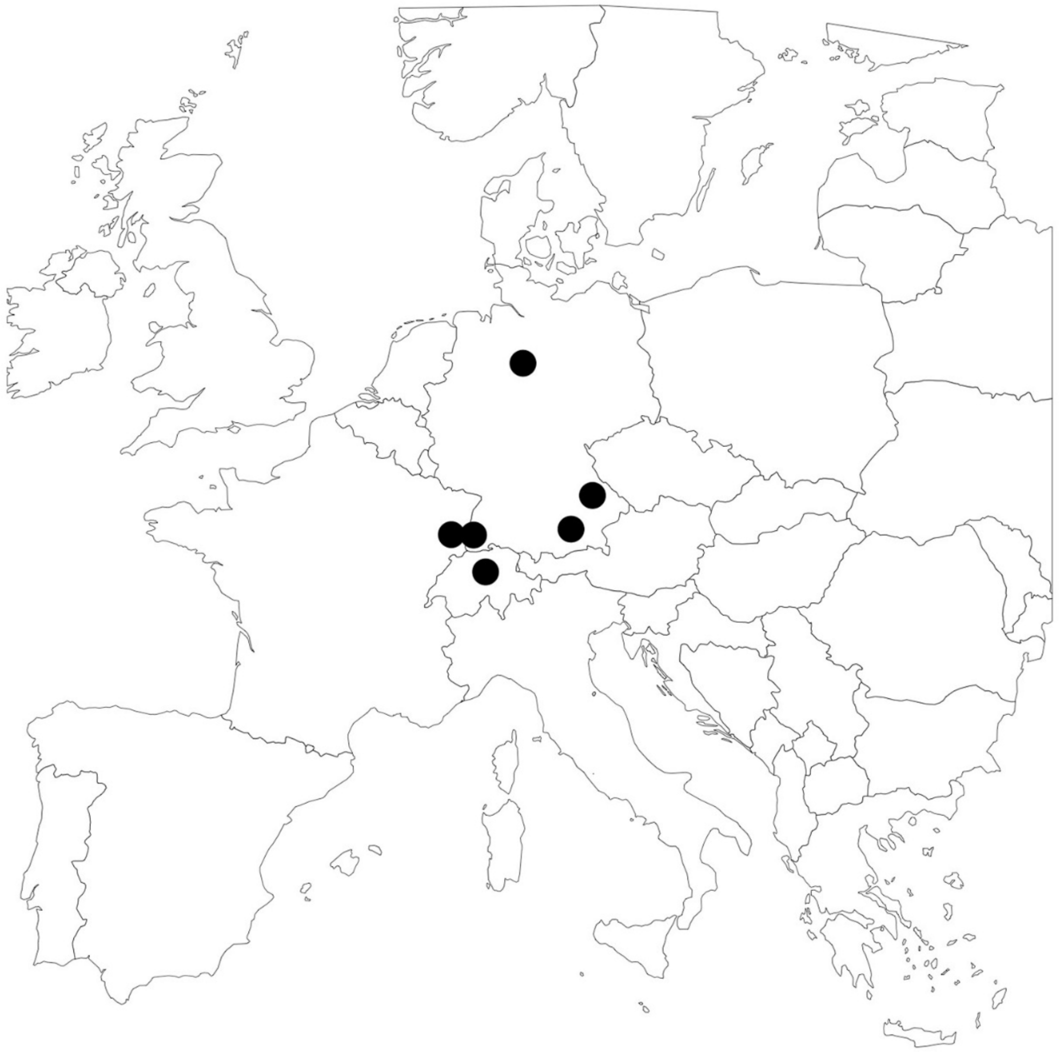

Appendix Figure 3. Geographic localization of all published human *Taenia martis* infections (*3*–*7*).

References

1. Neumayr A. Antiparasitic treatment recommendations: a practical guide to clinical parasitology. 2018 [cited 2022 Dec 18]. https://edoc.unibas.ch/69153

2. Mueller A, Förch G, Zustin J, Muntau B, Schuldt G, Tappe D. Case report: molecular identification of larval *Taenia martis* infection in the pouch of Douglas. Am J Trop Med Hyg. 2020;103:2315–7. PubMed https://doi.org/10.4269/ajtmh.20-0782

3. Deplazes P, Eichenberger RM, Grimm F. Wildlife-transmitted *Taenia* and *Versteria* cysticercosis and coenurosis in humans and other primates. Int J Parasitol Parasites Wildl. 2019;9:342–58. PubMed https://doi.org/10.1016/j.ijppaw.2019.03.013

4. Eberwein P, Haeupler A, Kuepper F, Wagner D, Kern WV, Muntau B, et al. Human infection with marten tapeworm. Emerg Infect Dis. 2013;19:1152–4. PubMed https://doi.org/10.3201/eid1907.121114

5. Brunet J, Benoilid A, Kremer S, Dalvit C, Lefebvre N, Hansmann Y, et al. First case of human cerebral *Taenia martis* cysticercosis. J Clin Microbiol. 2015;53:2756–9. PubMed https://doi.org/10.1128/JCM.01033-15

6. Koch T, Schoen C, Muntau B, Addo M, Ostertag H, Wiechens B, et al. Molecular diagnosis of human *Taenia martis* eye infection. Am J Trop Med Hyg. 2016;94:1055–7. PubMed https://doi.org/10.4269/ajtmh.15-0881

7. Rudelius M, Brehm K, Poelcher M, Spinner C, Rosenwald A, da Costa CP. First case of human peritoneal cysticercosis mimicking peritoneal carcinosis: necessity of laparoscopy and histologic assessment for the correct diagnosis. JMM Case Rep. 2017;4:e005097. PubMed https://doi.org/10.1099/jmmcr.0.005097
